# Supplementary material for: Operational feasibility of the ultra-portable digital X-rays with Computer-Aided Detection (CAD) for community active case finding for TB in Nigeria: Health care workers and client’s perspectives
Source: PLOS Glob Public Health. 2025 Oct 22;5(10):e0005234. doi: 10.1371/journal.pgph.0005234 (PMC12543118; doi:10.1371/journal.pgph.0005234)
Supplement: S1 Text — (DOCX) [file pgph.0005234.s001.docx]

**RESPONDENTS (RADIOGRAPHER'S) STATE_UPDX**

|  | | Frequency | Percent | Valid Percent | Cumulative Percent |
| --- | --- | --- | --- | --- | --- |
| Valid | Benue | 1 | 10.0 | 10.0 | 10.0 |
|  | Cross River | 1 | 10.0 | 10.0 | 20.0 |
|  | Delta | 1 | 10.0 | 10.0 | 30.0 |
|  | Kano_DLB2 | 1 | 10.0 | 10.0 | 40.0 |
|  | Kano_DLB6 | 1 | 10.0 | 10.0 | 50.0 |
|  | Katsina | 1 | 10.0 | 10.0 | 60.0 |
|  | Nasarawa | 1 | 10.0 | 10.0 | 70.0 |
|  | Osun_PDX1 | 1 | 10.0 | 10.0 | 80.0 |
|  | Osun_PDX2 | 1 | 10.0 | 10.0 | 90.0 |
|  | Oyo | 1 | 10.0 | 10.0 | 100.0 |
|  | Total | 10 | 100.0 | 100.0 |  |

| **AGE** | | | | | |
| --- | --- | --- | --- | --- | --- |
|  | | Frequency | Percent | Valid Percent | Cumulative Percent |
| Valid |  |  |  |  |  |
|  | 29 | 2 | 20.0 | 20.0 | 20.0 |
|  | 30 | 2 | 20.0 | 20.0 | 40.0 |
|  | 31 | 1 | 10.0 | 10.0 | 50.0 |
|  | 33 | 1 | 10.0 | 10.0 | 60.0 |
|  | 34 | 2 | 20.0 | 20.0 | 80.0 |
|  | 38 | 1 | 10.0 | 10.0 | 90.0 |
|  | 45 | 1 | 10.0 | 10.0 | 100.0 |
|  | Total | 10 | 100.0 | 100.0 |  |

| **SEX** | | | | | |
| --- | --- | --- | --- | --- | --- |
|  | | Frequency | Percent | Valid Percent | Cumulative Percent |
| Valid | Male | 9 | 90.0 | 90.0 | 90.0 |
|  | Female | 1 | 10.0 | 10.0 | 100.0 |
|  | Total | 10 | 100.0 | 100.0 |  |
|  |  |  |  |  |  |

| 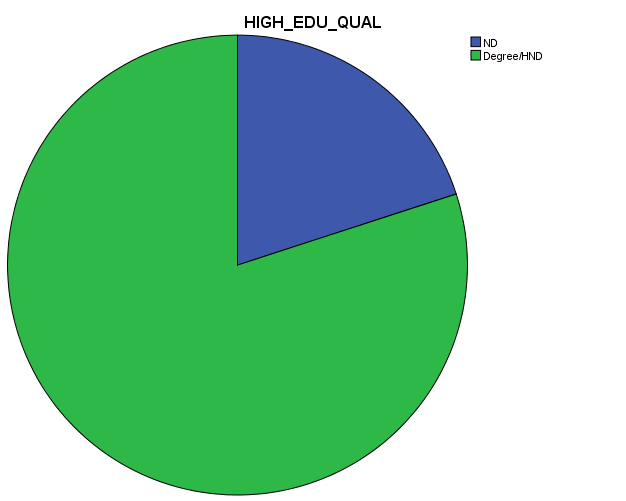  **HIGH_EDU_QUAL** | | | | | |
| --- | --- | --- | --- | --- | --- |
|  | | | | | |
|  | | Frequency | Percent | Valid Percent | Cumulative Percent |
| Valid | ND | 2 | 20.0 | 20.0 | 20.0 |
|  | Degree/HND | 8 | 80.0 | 80.0 | 100.0 |
|  | Total | 10 | 100.0 | 100.0 |  |

| **NO_YRS_RADIOGR** | | | | | |
| --- | --- | --- | --- | --- | --- |
|  | | Frequency | Percent | Valid Percent | Cumulative Percent |
| Valid | 1-4 | 3 | 30.0 | 30.0 | 30.0 |
|  | 5-9 | 5 | 50.0 | 50.0 | 80.0 |
|  | 10-14 | 1 | 10.0 | 10.0 | 90.0 |
|  | 15-20 | 1 | 10.0 | 10.0 | 100.0 |
|  | Total | 10 | 100.0 | 100.0 |  |

| **NO_YRS_TB PROG** | | | | | |
| --- | --- | --- | --- | --- | --- |
|  | | Frequency | Percent | Valid Percent | Cumulative Percent |
| Valid | Below 4 years | 8 | 80.0 | 80.0 | 80.0 |
|  | 5-9 years | 2 | 20.0 | 20.0 | 100.0 |
|  | Total | 10 | 100.0 | 100.0 |  |

| **PREV EXPER_CAD** | | | | | |
| --- | --- | --- | --- | --- | --- |
|  | | Frequency | Percent | Valid Percent | Cumulative Percent |
| Valid | No | 6 | 60.0 | 60.0 | 60.0 |
|  | Yes | 4 | 40.0 | 40.0 | 100.0 |
|  | Total | 10 | 100.0 | 100.0 |  |

| **EASE OF ASSEM_SET UP** | | | | | |
| --- | --- | --- | --- | --- | --- |
|  | | Frequency | Percent | Valid Percent | Cumulative Percent |
| Valid | Strongly Agree | 10 | 100.0 | 100.0 | 100.0 |

| **EASE OF OP_USER FRI** | | | | | |
| --- | --- | --- | --- | --- | --- |
|  | | Frequency | Percent | Valid Percent | Cumulative Percent |
| Valid | Agree | 6 | 60.0 | 60.0 | 60.0 |
|  | Strongly Agree | 4 | 40.0 | 40.0 | 100.0 |
|  | Total | 10 | 100.0 | 100.0 |  |
| **PROFICI** | | | | | |
|  | | Frequency | Percent | Valid Percent | Cumulative Percent |
| Valid | Strongly Agree | 10 | 100.0 | 100.0 | 100.0 |

| **SET UP_TIME** | | | | | |
| --- | --- | --- | --- | --- | --- |
|  | | Frequency | Percent | Valid Percent | Cumulative Percent |
| Valid | 3 mins | 1 | 10.0 | 10.0 | 10.0 |
|  | 5 mins | 1 | 10.0 | 10.0 | 20.0 |
|  | 5-10 mins | 4 | 40.0 | 40.0 | 60.0 |
|  | 7 mins | 3 | 30.0 | 30.0 | 90.0 |
|  | 20 mins | 1 | 10.0 | 10.0 | 100.0 |
|  | Total | 10 | 100.0 | 100.0 |  |

| **Aside from the UPDX staff, How many persons on the average do you need to support commencement of screening activity at a site?** | | | | | |
| --- | --- | --- | --- | --- | --- |
|  | | Frequency | Percent | Valid Percent | Cumulative Percent |
| Valid | None | 3 | 30.0 | 30.0 | 30.0 |
|  | 1 | 1 | 10.0 | 10.0 | 40.0 |
|  | 2 | 4 | 40.0 | 40.0 | 80.0 |
|  | 3 | 2 | 20.0 | 20.0 | 100.0 |
|  | Total | 10 | 100.0 | 100.0 |  |

| **On the average, how many persons can you screen daily using the UPDX? 70 persons** | | | | | |
| --- | --- | --- | --- | --- | --- |
|  | | Frequency | Percent | Valid Percent | Cumulative Percent |
| Valid | 60 | 1 | 10.0 | 10.0 | 10.0 |
|  | 70 | 1 | 10.0 | 10.0 | 20.0 |
|  | 88 | 4 | 40.0 | 40.0 | 60.0 |
|  | >100 | 1 | 10.0 | 10.0 | 70.0 |
|  | 200 | 3 | 30.0 | 30.0 | 100.0 |
|  | Total | 10 | 100.0 | 100.0 |  |

| **On the average how many hours/day do you spend out in the field screening clients, 8 Hours** | | | | | |
| --- | --- | --- | --- | --- | --- |
|  | | Frequency | Percent | Valid Percent | Cumulative Percent |
| Valid | 4 | 3 | 30.0 | 30.0 | 30.0 |
|  | 5 | 3 | 30.0 | 30.0 | 60.0 |
|  | 8 | 4 | 40.0 | 40.0 | 100.0 |
|  | Total | 10 | 100.0 | 100.0 |  |

| **Can you estimate how long (How many days or weeks of operations) it took you to get comfortable/proficient with operating the machine? 2 months** | | | | | |
| --- | --- | --- | --- | --- | --- |
|  | | Frequency | Percent | Valid Percent | Cumulative Percent |
| Valid | 1 | 2 | 20.0 | 20.0 | 20.0 |
|  | 2 | 7 | 70.0 | 70.0 | 90.0 |
|  | 3 | 1 | 10.0 | 10.0 | 100.0 |
|  | Total | 10 | 100.0 | 100.0 |  |

| **The image quality of the UPDX is not different from a normal CXR quality?** | | | | | |
| --- | --- | --- | --- | --- | --- |
|  | | Frequency | Percent | Valid Percent | Cumulative Percent |
| Valid | Strongly Disagree | 1 | 10.0 | 10.0 | 10.0 |
|  | Neutral | 3 | 30.0 | 30.0 | 40.0 |
|  | Agree | 3 | 30.0 | 30.0 | 70.0 |
|  | Strongly Agree | 3 | 30.0 | 30.0 | 100.0 |
|  | Total | 10 | 100.0 | 100.0 |  |

| **Transfer of CXR images from the UPDX system to another device is easy?** | | | | | |
| --- | --- | --- | --- | --- | --- |
|  | | Frequency | Percent | Valid Percent | Cumulative Percent |
| Valid | Neutral | 1 | 10.0 | 10.0 | 10.0 |
|  | Agree | 4 | 40.0 | 40.0 | 50.0 |
|  | Strongly Agree | 5 | 50.0 | 50.0 | 100.0 |
|  | Total | 10 | 100.0 | 100.0 |  |

| **How long (secs) does it take to transfer images wirelessly from the detector to the laptop for processing? 3 mins** | | | | | | | | | | | | | | | |
| --- | --- | --- | --- | --- | --- | --- | --- | --- | --- | --- | --- | --- | --- | --- | --- |
|  | | | | Frequency | | | | Percent | | Valid Percent | | | Cumulative Percent | | |
| Valid | 10 seconds | | | 1 | | | | 10.0 | | 10.0 | | | 10.0 | | |
|  | 30 seconds | | | 3 | | | | 30.0 | | 30.0 | | | 40.0 | | |
|  | Less than one minute | | | 1 | | | | 10.0 | | 10.0 | | | 50.0 | | |
|  | One minute | | | 2 | | | | 20.0 | | 20.0 | | | 70.0 | | |
|  | 120 seconds | | | 1 | | | | 10.0 | | 10.0 | | | 80.0 | | |
|  | 3-5 minutes | | | 2 | | | | 20.0 | | 20.0 | | | 100.0 | | |
|  | Total | | | 10 | | | | 100.0 | | 100.0 | | |  | | |
| **Adapting the device settings (exposure time, power settings) for the UPDX Generator to the different sizes of clients screened is easy?** | | | | | | | | | | | | | | |  |
|  | | | Frequency | | | Percent | | | Valid Percent | | | Cumulative Percent | | |  |
| Valid | Disagree | | 1 | | | 10.0 | | | 10.0 | | | 10.0 | | |  |
|  | Neutral | | 1 | | | 10.0 | | | 10.0 | | | 20.0 | | |  |
|  | Agree | | 7 | | | 70.0 | | | 70.0 | | | 90.0 | | |  |
|  | Strongly Agree | | 1 | | | 10.0 | | | 10.0 | | | 100.0 | | |  |
|  | Total | | 10 | | | 100.0 | | | 100.0 | | |  | | |  |
| **A fully charged Generator is adequate for screening clients through-out working the day** | | | | | | | | | | | | | | |  |
|  | | | Frequency | | | Percent | | | Valid Percent | | | Cumulative Percent | | |  |
| Valid | Disagree | | 3 | | | 30.0 | | | 30.0 | | | 30.0 | | |  |
|  | Neutral | | 1 | | | 10.0 | | | 10.0 | | | 40.0 | | |  |
|  | Agree | | 4 | | | 40.0 | | | 40.0 | | | 80.0 | | |  |
|  | Strongly Agree | | 2 | | | 20.0 | | | 20.0 | | | 100.0 | | |  |
|  | Total | | 10 | | | 100.0 | | | 100.0 | | |  | | |  |
| **I often experience technical challenges with the UPDX system** | | | | | | | | | | | | | |  |  |
|  | | Frequency | | | Percent | | Valid Percent | | | | Cumulative Percent | | |  |  |
| Valid | Disagree | 7 | | | 70.0 | | 70.0 | | | | 70.0 | | |  |  |
|  | Agree | 3 | | | 30.0 | | 30.0 | | | | 100.0 | | |  |  |
|  | Total | 10 | | | 100.0 | | 100.0 | | | |  | | |  |  |

| **Do you think the device is portable enough for 1 person to carry?** | | | | | |
| --- | --- | --- | --- | --- | --- |
|  | | Frequency | Percent | Valid Percent | Cumulative Percent |
| Valid | Strongly Disagree | 9 | 90.0 | 90.0 | 90.0 |
|  | Disagree | 1 | 10.0 | 10.0 | 100.0 |
|  | Total | 10 | 100.0 | 100.0 |  |

| **The UPDX power source is adequate for providing power through-out the day for screening clients?** | | | | | |
| --- | --- | --- | --- | --- | --- |
|  | | Frequency | Percent | Valid Percent | Cumulative Percent |
| Valid | Neutral | 4 | 40.0 | 40.0 | 40.0 |
|  | Agree | 2 | 20.0 | 20.0 | 60.0 |
|  | Strongly Agree | 4 | 40.0 | 40.0 | 100.0 |
|  | Total | 10 | 100.0 | 100.0 |  |

| **What power source do you usually use on the field while screening?** | | | | | |
| --- | --- | --- | --- | --- | --- |
|  | | Frequency | Percent | Valid Percent | Cumulative Percent |
| Valid | Mobisum Solar Panel | 10 | 100.0 | 100.0 | 100.0 |

| **How many exposures on the average can you capture on a fully charged battery?** | | | | | |
| --- | --- | --- | --- | --- | --- |
|  | | Frequency | Percent | Valid Percent | Cumulative Percent |
| Valid | 60 | 1 | 10.0 | 10.0 | 10.0 |
|  | 70 | 2 | 20.0 | 20.0 | 30.0 |
|  | 80 | 3 | 30.0 | 30.0 | 60.0 |
|  | 100 | 1 | 10.0 | 10.0 | 70.0 |
|  | 120 | 1 | 10.0 | 10.0 | 80.0 |
|  | 130 | 1 | 10.0 | 10.0 | 90.0 |
|  | 200 | 1 | 10.0 | 10.0 | 100.0 |
|  | Total | 10 | 100.0 | 100.0 |  |

| **Conducting daily maintenance on the machine is easy** | | | | | |
| --- | --- | --- | --- | --- | --- |
|  | | Frequency | Percent | Valid Percent | Cumulative Percent |
| Valid | Disagree | 5 | 50.0 | 50.0 | 50.0 |
|  | Neutral | 2 | 20.0 | 20.0 | 70.0 |
|  | Agree | 2 | 20.0 | 20.0 | 90.0 |
|  | Strongly Agree | 1 | 10.0 | 10.0 | 100.0 |
|  | Total | 10 | 100.0 | 100.0 |  |

| **The radiation safety profile of the UPDX is satisfactory?** | | | | | |
| --- | --- | --- | --- | --- | --- |
|  | | Frequency | Percent | Valid Percent | Cumulative Percent |
| Valid | Strongly Disagree | 1 | 10.0 | 10.0 | 10.0 |
|  | Disagree | 1 | 10.0 | 10.0 | 20.0 |
|  | Neutral | 1 | 10.0 | 10.0 | 30.0 |
|  | Agree | 6 | 60.0 | 60.0 | 90.0 |
|  | Strongly Agree | 1 | 10.0 | 10.0 | 100.0 |
|  | Total | 10 | 100.0 | 100.0 |  |

| **The radiation safety measures provided for your protection adequate?** | | | | | |
| --- | --- | --- | --- | --- | --- |
|  | | Frequency | Percent | Valid Percent | Cumulative Percent |
| Valid | o | 1 | 10.0 | 10.0 | 10.0 |
|  | Strongly Disagree | 2 | 20.0 | 20.0 | 30.0 |
|  | Disagree | 4 | 40.0 | 40.0 | 70.0 |
|  | Neutral | 1 | 10.0 | 10.0 | 80.0 |
|  | Agree | 2 | 20.0 | 20.0 | 100.0 |
|  | Total | 10 | 100.0 | 100.0 |  |

| **The training I received for the UPDX operation was adequate and sufficient to enable me successfully carry out daily operations with the machine.** | | | | | |
| --- | --- | --- | --- | --- | --- |
|  | | Frequency | Percent | Valid Percent | Cumulative Percent |
| Valid | Neutral | 2 | 20.0 | 20.0 | 20.0 |
|  | Agree | 3 | 30.0 | 30.0 | 50.0 |
|  | Strongly Agree | 5 | 50.0 | 50.0 | 100.0 |
|  | Total | 10 | 100.0 | 100.0 |  |
| **The quality of post-training technical support (remote and physical) I receive is adequate?** | | | | | |
|  | | Frequency | Percent | Valid Percent | Cumulative Percent |
| Valid | Disagree | 1 | 10.0 | 10.0 | 10.0 |
|  | Agree | 5 | 50.0 | 50.0 | 60.0 |
|  | Strongly Agree | 4 | 40.0 | 40.0 | 100.0 |
|  | Total | 10 | 100.0 | 100.0 |  |

| **I find it easy reaching the DELFT support staff and getting the required assistance when in need of support on the field?** | | | | | |
| --- | --- | --- | --- | --- | --- |
|  | | Frequency | Percent | Valid Percent | Cumulative Percent |
| Valid | Agree | 1 | 10.0 | 10.0 | 10.0 |
|  | Strongly Agree | 9 | 90.0 | 90.0 | 100.0 |
|  | Total | 10 | 100.0 | 100.0 |  |

|  |  |  |  |  |  |  |  |  |
| --- | --- | --- | --- | --- | --- | --- | --- | --- |
