# Supplementary material for: Operational feasibility of the ultra-portable digital X-rays with Computer-Aided Detection (CAD) for community active case finding for TB in Nigeria: Health care workers and client’s perspectives
Source: PLOS Glob Public Health. 2025 Oct 22;5(10):e0005234. doi: 10.1371/journal.pgph.0005234 (PMC12543118; doi:10.1371/journal.pgph.0005234)
Supplement: S2 Text — (DOCX) [file pgph.0005234.s002.docx]

**Annex 1: Study Instruments**

**Questionnaire for HCW: End-Users (Radiographers)**

Respondent sociodemographic data

Initials__________________ Age ______ Sex _______ State ________________________

Highest Educational Qualification___________________

Number of years of practice as a Radiographer_______________________

Number of years of engagement in the TB program ________________

Previous experience with CAD? Yes/No

| No. | DOMAIN | ITEM | **Strongly Disagree** | **Disagree** | **Neutral** | **Agree** | **Strongly Agree** |
| --- | --- | --- | --- | --- | --- | --- | --- |
| 1 | **Initiation of UPDX screening** | The UPDX machine and accessories/components are quite easy to assemble and set-up? | **Strongly Disagree** | **Disagree** | **Neutral** | **Agree** | **Strongly Agree** |
| 2 | **Initiation of UPDX screening** | The programs and software on the laptops/tablets are user-friendly and easy to operate for entering and editing patient details/data and retrieving results. | **Strongly Disagree** | **Disagree** | **Neutral** | **Agree** | **Strongly Agree** |
| 3 | **Initiation of UPDX screening** | I currently consider myself proficient with operating the UPDX system machine?  Can you explain why___________________________________________ | **Strongly Disagree** | **Disagree** | **Neutral** | **Agree** | **Strongly Agree** |
| 4 | **Initiation of UPDX screening** | How long on the average does it take for the UPDX system to be set up for screening at a location______________ |  |  |  |  |  |
| 5 | **Initiation of UPDX screening** | Aside from the UPDX staff, How many persons on the average do you need to support commencement of screening activity at a site ______________________ |  |  |  |  |  |
| 6 | **Initiation of UPDX screening** | On the average, how many persons can you screen daily using the UPDX? ___________ |  |  |  |  |  |
| 7 | **Initiation of UPDX screening** | On the average how many hours/day do you spend out in the field screening clients  _______________________ |  |  |  |  |  |
| 8 | **Initiation of UPDX screening** | Can you estimate how long (How many days or weeks of operations) it took you to get comfortable/proficient with operating the machine? ________________ |  |  |  |  |  |
| 9 | **Technical Operations** | The image quality of the UPDX is not different from a normal CXR quality? | **Strongly Disagree** | **Disagree** | **Neutral** | **Agree** | **Strongly Agree** |
| 10 | **Technical Operations** | Transfer of CXR images from the UPDX system to another device is easy? | **Strongly Disagree** | **Disagree** | **Neutral** | **Agree** | **Strongly Agree** |
| 11 | **Technical Operations** | How long (secs) does it take to transfer images wirelessly from the detector to the laptop for processing?_______ |  |  |  |  |  |
| 12 | **Technical Operations** | What is your preferred deployment method for using CAD /AI technology to interpret chest x-rays?  Choose one.   1. Offline (using local hardware) without connection to Internet 2. Online (using cloud method) with connection to Internet 3. Hybrid: using locally installed CAD to parse X-ray offline and backup / synchronization when there’s internet connection or on demand. |  |  |  |  |  |
| 13 | **Technical Operations** | Adapting the device settings (exposure time, power settings) for the UPDX Generator to the different sizes of clients screened is easy?  Additional comments______________ | **Strongly Disagree** | **Disagree** | **Neutral** | **Agree** | **Strongly Agree** |
| 14 | **Technical Operations** | A fully charged Generator is adequate for screening clients through-out working the day | **Strongly Disagree** | **Disagree** | **Neutral** | **Agree** | **Strongly Agree** |
| 15 | **Technical Operations** | I often experience technical challenges with the UPDX system | **Strongly Disagree** | **Disagree** | **Neutral** | **Agree** | **Strongly Agree** |
| 16 | **Technical Operations** | How often do you experience technical challenges with the UPDX system? Choose one.   1. Daily 2. At least 2 times weekly 3. At least 3 times weekly 4. Weekly 5. Others (Explain)___________ |  |  |  |  |  |
| 17 | **Technical Operations** | What are the top 2 technical problems you have experienced while using this machine?  1.  2. |  |  |  |  |  |
| 18 | **Technical Operations** | Which one of the challenges above is the commonest challenge you encounter with the UPDX system? ________ |  |  |  |  |  |
| 19 | **Technical Operations** | Do you think the device is portable enough for 1 person to carry? | **Strongly Disagree** | **Disagree** | **Neutral** | **Agree** | **Strongly Agree** |
| 20 | **Power** | The UPDX power source is adequate for providing power through-out the day for screening clients? | **Strongly Disagree** | **Disagree** | **Neutral** | **Agree** | **Strongly Agree** |
| 21 | **Power** | What power source do you usually use on the field while screening?  _______________________ |  |  |  |  |  |
| 22 | **Power** | How long does your battery power last when you are on the field?  _____________________ |  |  |  |  |  |
| 23 | **Power** | How many exposures on the average can you capture on a fully charged battery?  ____________________ |  |  |  |  |  |
| 24 | **Maintenance and safety** | Conducting daily maintenance on the machine is easy | **Strongly Disagree** | **Disagree** | **Neutral** | **Agree** | **Strongly Agree** |
| 25 | **Maintenance and safety** | The radiation safety profile of the UPDX is satisfactory? | **Strongly Disagree** | **Disagree** | **Neutral** | **Agree** | **Strongly Agree** |
| 26 | **Maintenance and safety** | The radiation safety measures provided for your protection adequate?  If disagree, why____________________ | **Strongly Disagree** | **Disagree** | **Neutral** | **Agree** | **Strongly Agree** |
| 27 | **Training and support** | The training I received for the UPDX operation was adequate and sufficient to enable me successfully carry out daily operations with the machine.    N/B If not adequate, What areas do you think would require further training? ________________________________ | **Strongly Disagree** | **Disagree** | **Neutral** | **Agree** | **Strongly Agree** |
| 28 | **Training and support** | The quality of post-training technical support (remote and physical) I receive is adequate? | **Strongly Disagree** | **Disagree** | **Neutral** | **Agree** | **Strongly Agree** |
| 29 | **Training and support** | I find it easy reaching the DELFT support staff and getting the required assistance when in need of support on the field? | **Strongly Disagree** | **Disagree** | **Neutral** | **Agree** | **Strongly Agree** |
| 30 | **Training and support** | What would you want to be improved on the DLB system to make your work easier?  _______________________ _______________________  _______________________ | **Strongly Disagree** | **Disagree** | **Neutral** | **Agree** | **Strongly Agree** |

**Questionnaire for Key Informants – IPs Project Managers, NTP NC**

Respondent sociodemographic data

Initials__________________ Age ______ Sex _______ State ____________________

Highest Educational Qualification___________________

Number of years of engagement in the TB program ________________

1. Can you briefly describe your role in CXR with CAD screening activities
2. What is your impression about the TB burden in Nigeria and the effectiveness of strategies to find the missing TB cases?
3. Are you aware of the WHO recommendation on use of CXR for TB diagnosis?
4. What is your perspective about the use of X-ray in TB diagnosis?
5. What is your opinion about the use of Ultra Portable digital X-rays and Artificial Intelligence (CAD) in TB case finding in Nigeria?
6. In your view, what are the benefits of rolling out this technology in Nigeria?
   - 1. Are there any drawbacks?
7. Do you know if there are guidelines that support the use of UPDX and the CAD in TB case finding in Nigeria?
   - 1. If Yes, can you tell me how you used the guidelines and if it provides sufficient information to users?
8. From your experience, what are the implications of implementing the UPDX and CAD for Case finding:
   - 1. Prompt answers for information on Implementation Challenges, operational costs, efficiency of the intervention and return on investment
9. In your opinion what factors would act as enablers to a successful implementation of the UPDX with CAD?
   - 1. How do you think these enablers can be encouraged?
10. What would you consider barriers to implementation of this technology ?
    - 1. How could these barriers be overcome?

**Questionnaire for Radiologists engaged for Clinical review of Xrays from UPDX**

Respondent sociodemographic data

Initials__________________ Age ______ Sex _______ State ________________

Highest Educational Qualification___________________

Number of years of practice as a Radiologist_______________________

Number of years of engagement in the TB program ________________

1. Can you briefly describe your role in the UPDX and CAD screening activities?
2. What is your impression about the TB burden in Nigeria?
3. Did your impression of the burden of TB in Nigeria change after you started engagement with the TB program?
4. What is your perspective about the use of X-ray in TB diagnosis?
5. What can you tell us about the quality of film from the Ultraportable digital X-rays compared to the stationary X-rays in the facilities.
6. What operational advantages/dis-advantages have you observed with the UPDX over the conventional X-ray machines
7. What is your opinion about the use of Ultraportable digital X-rays and Artificial Intelligence (CAD) in TB case finding in Nigeria?
   - 1. What do you see as the benefits and potential disadvantages?
     2. Do you trust the result of CAD? Why / why not?
8. How easy do you find CAD output to interpret?
   - 1. Is it useful to your work?
9. As the end user, what advice will you give to the manufacturers to make the UPDX more efficient in TB service delivery

**Questionnaire for HCWs (LGTBLS, DOTS)**

Respondent sociodemographic data

Initials__________________ Age ______ Sex _______

Occupation_____________________ State ______________

Highest Educational Qualification_______________________

Number of years of engagement/experience in the TB program ________________

A. Warm-up / Introduction

Can you tell me about how a typical day working with the Ultraportable Digital X ray team goes?

(PROMPT: What preparatory steps are taken, Where are the tests performed, Which HCWs perform the tests, how are you involved?)

What has been your experience so far while using the Portable Digital X ray with Artificial Intelligence for ACF in your facility or LGA?

B. Perception of Tests

1. How has your practice (TB case finding services/activities) been affected by the introduction of the new Ultraportable Digital X ray with Artificial Intelligence systems for screening?

(PROMPT: How has it affected your workload? Do you think you are more or less busy since the PDX was used/introduced?)

2. How well do you think the Ultraportable Digital X ray with Artificial Intelligence intervention has been successfully integrated into the TB program ACF strategy?

3. Can you tell me about any challenges/problems experienced while using the Ultraportable Digital X ray with Artificial Intelligence for ACF activities?

(PROMPT: Can you tell me about the most challenging/difficult time you had when using the UPDX with CAD in ACF activities? Can you tell me about the best or any particularly good time you had when using new UPDX systems)

4. Since the UPDX with CAD was introduced here for ACF activities, can you tell me about any benefits HCWs may have experienced from its use if any?

(PROMPT: How have they helped HCWs? What aspects of your work have become easier? Which HCWs in particular have been affected?)

5. Can you describe any problems/disadvantages or challenges created by the UPDX for HCWs or TB program?

(PROMPT: If yes, can you tell me about them? What are the challenges for the HCWs? What aspects of your work has become more difficult? Which HCWs in particular have been affected in this way? How do they deal with these difficulties/challenges?)

C. Knowledge / Training

6. Before the UPDX with CAD was used for ACF in your Community or LGA, did you receive any training/orientation on how it will be used and how you will work with the UPDX team?

(PROMPT: If Yes, please describe the training received: by whom, where, how long? What your experience of this training or orientation; how did you find it? Were there any good or bad things about it? How did you think orientation differs from training? Is there anything you would change in the way you were trained on working with the PDX? If you were designing a training programme for HCWs in another district how would you recommend it should be done?)

7. Can you describe for me exactly how you work with the UPDX/CAD team for ACF?

(PROMPT: What are the steps you follow? Do you follow any guideline or SOP for this process?)

D. Social Environment/Acceptability

8. In your opinion, how do your patients feel about the screening with UPDX with CAD

(PROMPT: Acceptability of the screening test/procedure - Positive or Negative?

9. From your perspective and experience with the implementation, How do you think patients/clients have been affected by the introduction of the new UPDX with CAD for ACF?

(PROMPT: What have been the benefits or advantages for patients? Have there been any difficulties from a patient point of view? How have patients responded to the introduction of the PDX with CAD?)

10. Considering the definitive results for diagnosis are usually not ready the same day, how do your patients diagnosed either clinically or bacteriologically respond to the results when available?

11. What is your experience on contacts tracing for patients who are diagnosed with TB (bacteriologically or clinically) through UPDX/CAD ACF intervention?

(PROMPT: Is it being done? How is it done in this facility? Have there been any difficulties with contact tracing. Can you tell me about the most difficult time you had with contact tracing for a diagnosed TB case from the UPDX/CAD intervention. How did you deal with that? Have you been able to discover more cases from contact tracing of diagnosed TB cases from the UPDX/CAD intervention?)

12. Do you think that the UPDX with CAD should continue to be used for TB screening in your facility/Community/LGA? Yes or No

(PROMPT: If Yes or No, what is the most important reason why you think this? Is it Reliability of results, Workload?, Ease/Difficulty of testing?)

E. Supervision

13. Since UPDX/CAD screening was introduced in this LGA/State, can you tell me about any supervisory visits you have experienced during its use in your community?

(If Yes, PROMPT: who visited? what feedback did you receive after the supervision?)

F. Future of UPDX/CAD Programme

14. Can you think of any barriers or challenges to the increased use of UPDX/CAD for ACF screening intervention in communities?

15. Can you think of factors that would facilitate or encourage the increased use of UPDX/CAD for ACF screening intervention in communities?

**Questionnaire for Patients – Exit interview**

**Respondent sociodemographic data**

Initials__________________ Age ______

Sex _______ State _____________________

Occupation_________________

Highest Educational Qualification_________________

**Knowledge and perceptions of TB;**

1. What do you know/can you tell me about Tuberculosis,

*Probe/Follow-on Question: Do you think somebody can have it and not know? How?*

**Willingness to be screened with PDX_CAD:**

2. How did you come to access XRay screening for TB?

3. How easy was it for you to access the XRay screening?

(Prompt: how long was the journey, were the timings convenient)

4. How did you feel about being screened for TB using the Ultraportable Xray machine or did you have some concerns about conducting the screening using the Xray?

*Probe/Follow-on Question: Can you explain reasons why you felt this way?*

5. Do you think you would be willing to recommend to your family and friends to come and conduct this screening tests using the Ultraportable digital X ray machine?

*Probe/Follow-on Question: Can you explain reasons why?*

**Belief/Confidence in result of screening/test:**

6. Do you know that your scan was interpreted by a computer device using artificial intelligence?

7. Do you believe or trust that this machine will be able to help detect TB disease if you or anybody else here has it.

*Probe/Follow-on Question: Why do you think so?*

*If they do not trust the results: would anything reassure you of its accuracy?*

*8.* Would you have preferred any other means other than this machine for testing if you have TB?

**Willingness to wait for result:**

9. Considering you are only able to get a preliminary Xray result now but not the final/confirmatory result from the Xray and sputum test today, how long are you willing to wait for the final result?

*Probe/Follow-on Question: Can you explain reasons why?*
