# Supplementary material for: Operational feasibility of the ultra-portable digital X-rays with Computer-Aided Detection (CAD) for community active case finding for TB in Nigeria: Health care workers and client’s perspectives
Source: PLOS Glob Public Health. 2025 Oct 22;5(10):e0005234. doi: 10.1371/journal.pgph.0005234 (PMC12543118; doi:10.1371/journal.pgph.0005234)
Supplement: S3 Text — (DOCX) [file pgph.0005234.s003.docx]

**Annex 2: Informed Consent Form**

**Title of the research:** OPERATIONAL FEASIBILITY AND EASE-OF-USE OF THE ULTRAPORTABLE DIGITAL X-RAY WITH CAD FOR COMMUNITY ACTIVE CASE FINDING IN NIGERIA; HEALTH CARE WORKERS AND CLIENTS PERSPECTIVES

**Name and affiliation of the researcher of applicant**:

**Sponsor of research:** This study is sponsored by KNCV Tuberculosis Foundation Nigeria, IHV-Nigeria, and the National Tuberculosis Leprosy and Buruli Ulcer Control Program.

**Purpose of the research:** The purpose of this research is to find out the enablers and barriers to the effective implementation of the Ultraportable Digital CXR with CAD for ACF in Nigeria.

**The procedure of the research:** You will be interviewed by a research assistant for a few minutes to get your perspective on the implementation of the Ultraportable Digital CXR with CAD for ACF in Nigeria.

**Risks:** The study poses no risk to you. The research procedure is not expected to expose you to any physical or psychological harm. Your participation in this research will not cost you anything.

**Benefits:** Your responses will help with improving the quality of implementation of the Ultraportable digital CXR with CAD.

**Confidentiality:** All information collected in this study will be given code numbers and no name will be recorded. This cannot be linked to you in any way and your name or any identifier will not be used in any publication or reports from this study. As part of our responsibility to conduct this research properly, authorized officials from study institutions may have access to these records.

**Voluntariness:** Your participation in this research is entirely voluntary**.** The services you will receive will not be affected in any way in case you choose not to participate in the research. You are free to withdraw from the research at any time after signing this form but, note that some of the information you have already submitted cannot be removed anymore and will be used in reports and publications without your personal details mentioned.

**Due inducement(s):** You will not be paid any fees for participating in the research.

**What happens to research participants and communities when the research is over:** Outcomes and recommendations from the study results will be circulated widely as appropriate and posted in the hospital's notice board for your information.

**Statement of the person obtaining informed consent:**

I have fully explained this research to ____________________________________ and have given sufficient information, including about risks and benefits, to make an informed decision.

DATE: _____________________ SIGNATURE: _______________________________

NAME: ______________________________________________

**Statement of the person giving consent:**

I have read the description of the research or have had it translated into a language I understand. I have also talked it over with the health staff to my satisfaction.

DATE: _________________SIGNATURE/THUMB PRINT: ___________________

NAME: _____________________________________________

**Contact information of Research/Project Supervisor:**
