# Supplementary material for: Operational feasibility of the ultra-portable digital X-rays with Computer-Aided Detection (CAD) for community active case finding for TB in Nigeria: Health care workers and client’s perspectives
Source: PLOS Glob Public Health. 2025 Oct 22;5(10):e0005234. doi: 10.1371/journal.pgph.0005234 (PMC12543118; doi:10.1371/journal.pgph.0005234)
Supplement: S1 Data — (PDF) [file pgph.0005234.s004.pdf]

Taguette Codebook

Participant Role description

7 highlights

Impression of TB burden

7 highlights

Changes in impression of TB burden

6 highlights

Xray Role in TB diag

7 highlights

Film quality in comparison

9 highlights

Advantage.Operational

3 highlights

Disadvantage.Operational

2 highlights

Advantage

9 highlights

Trust level

4 highlights

Usefulness to work

7 highlights

Advice to manufacturers

7 highlights

Opinion on UPDX/CAD use in NIG

4 highlights

Ease of interpretation

6 highlights

Baseline knowledge of UPDX

1 highlight

Disadvantage

8 highlights
