# Supplementary material for: Operational feasibility of the ultra-portable digital X-rays with Computer-Aided Detection (CAD) for community active case finding for TB in Nigeria: Health care workers and client’s perspectives
Source: PLOS Glob Public Health. 2025 Oct 22;5(10):e0005234. doi: 10.1371/journal.pgph.0005234 (PMC12543118; doi:10.1371/journal.pgph.0005234)
Supplement: S2 Data — (PDF) [file pgph.0005234.s005.pdf]

Taguette Codebook

Suggest

0 highlights

Ease of use

2 highlights

Technical difficulties frequency

8 highlights

Commonest technical difficulties

8 highlights

Most common Tech difficulty

10 highlights

Power source

9 highlights

Suggest.Worforce strength

6 highlights

Suggest.Maintenance.Local availability Of

1 highlight

Suggest.Maintenance.Local parts

1 highlight

Suggest.Incentives

1 highlight

Suggest.Technical.Remote-adjustable detector pole

1 highlight

Suggest.Technical.Exposure knob on app

1 highlight

Suggest.Funding

1 highlight

Suggest.Awareness

1 highlight

Portability

3 highlights

Safety issues

4 highlights

Suggest.Safety

6 highlights

Suggest.Technical.System upgrade

1 highlight

Suggest.Wireless

3 highlights

CAD proficiency

3 highlights

Suggest.Technical.Detector stand

2 highlights

Suggest.Technical.For Accuracy

1 highlight

Suggest.Data analysis time

1 highlight

Suggest.Mobility

1 highlight
