# Supplementary material for: Operational feasibility of the ultra-portable digital X-rays with Computer-Aided Detection (CAD) for community active case finding for TB in Nigeria: Health care workers and client’s perspectives
Source: PLOS Glob Public Health. 2025 Oct 22;5(10):e0005234. doi: 10.1371/journal.pgph.0005234 (PMC12543118; doi:10.1371/journal.pgph.0005234)
Supplement: S3 Data — (PDF) [file pgph.0005234.s006.pdf]

Taguette Codebook

Typical day

21 highlights

Experience so far with UPDX

15 highlights

How practice affected

23 highlights

Integration success

16 highlights

Challenging times using UPDX

5 highlights

Benefits

30 highlights

Challenges

35 highlights

Best times using UPDX

5 highlights

Training for UPDX

17 highlights

Work routine.UPDX

13 highlights

Patient perception and impact

35 highlights

Patients response to results

15 highlights

Contact tracing

15 highlights

Recommendation on Use

19 highlights

Supervision

16 highlights

Barriers to increased use

16 highlights

Facilitators of increased use

16 highlights

Work routine.SOPs

13 highlights
