# Supplementary material for: Operational feasibility of the ultra-portable digital X-rays with Computer-Aided Detection (CAD) for community active case finding for TB in Nigeria: Health care workers and client’s perspectives
Source: PLOS Glob Public Health. 2025 Oct 22;5(10):e0005234. doi: 10.1371/journal.pgph.0005234 (PMC12543118; doi:10.1371/journal.pgph.0005234)
Supplement: S4 Data — (PDF) [file pgph.0005234.s007.pdf]

Taguette Codebook

TB knowledge

22 highlights

Route of Access to Screening

19 highlights

Ease of Access

22 highlights

Concerns about the screening

21 highlights

Willingness to recommend

20 highlights

Awareness that UPDX uses AI

19 highlights

Confidence in UPDX

18 highlights

Reason for Trusting the screen

15 highlights

Preference for other testing types

19 highlights

Willingness to wait for final results

20 highlights

Reason for wait willingness

13 highlights

If anything would reassure of accuracy

7 highlights

Reason for willingness to recommend

17 highlights
