# Supplementary material for: Operational feasibility of the ultra-portable digital X-rays with Computer-Aided Detection (CAD) for community active case finding for TB in Nigeria: Health care workers and client’s perspectives
Source: PLOS Glob Public Health. 2025 Oct 22;5(10):e0005234. doi: 10.1371/journal.pgph.0005234 (PMC12543118; doi:10.1371/journal.pgph.0005234)
Supplement: S5 Data — (PDF) [file pgph.0005234.s008.pdf]

Taguette Codebook

Role description

5 highlights

Effectiveness of TB casefinding

2 highlights

Awareness of WHO recommendation

4 highlights

Role of Xray in TB diag

12 highlights

Role of UPDX CAD in Nigeria

0 highlights

Benefit of roll out

0 highlights

Drawbacks

0 highlights

Knowledge of UPDX CAD guideline

5 highlights

Implications of deployment

0 highlights

Enablers of success

0 highlights

How to encourage enablers

0 highlights

Implementation barriers

0 highlights

Overcoming barriers

1 highlight

Impression of TB burden

4 highlights

Benefit of roll out.Case detection

4 highlights

Benefit of roll out.Treatment

1 highlight

Benefit of roll out.Case notification

1 highlight

Benefit of roll out.The reach of screening

2 highlights

Benefit of roll out.Portability advantage

2 highlights

Benefit of roll out.Nonspecialist care

1 highlight

Benefit of roll out.Cost saving

1 highlight

Benefit of roll out.Diagnosis speed

2 highlights

Benefit of roll out.Nonspecific benefits/Others

1 highlight

Drawbacks.Cost

1 highlight

Drawbacks.Limited machine deployment

1 highlight

Drawbacks.Maintenance

2 highlights

Drawbacks.Workforce lack

1 highlight

Drawbacks.Relating to diagnosis

2 highlights

Drawbacks.Connectivity

1 highlight

Drawbacks.Others

1 highlight

Effectiveness of TB casefinding.Limitations

1 highlight

Effectiveness of TB casefinding.Recommendations

1 highlight

Enablers of success.Worker motivation

1 highlight

Enablers of success.Worker training

3 highlights

How to encourage enablers.Bring more machines

1 highlight

How to encourage enablers.Maintenance

2 highlights

How to encourage enablers.Workforce motivation/incentives

3 highlights

Enablers of success.Machine availability

1 highlight

Enablers of success.Maintenance

4 highlights

Enablers of success.Workforce availability

2 highlights

Enablers of success.Others

3 highlights

How to encourage enablers.Increase workforce

2 highlights

How to encourage enablers.Increased Investment

2 highlights

How to encourage enablers.Others eg advocacy

1 highlight

Implementation barriers.Machine number

1 highlight

Implementation barriers.Cost

1 highlight

Implementation barriers.Workforce

2 highlights

Implementation barriers.Others

5 highlights

Implementation barriers.Mobility/Transport

1 highlight

Implementation barriers.Power supply

1 highlight

Implementation barriers.Refusal

0 highlights

Implications of deployment.Workforce

3 highlights

Implications of deployment.Quality assurance

1 highlight

Implications of deployment.Maintenance

1 highlight

Implications of deployment.Power issues

3 highlights

Implications of deployment.Mobility

2 highlights

Implications of deployment.Others

7 highlights

Implications of deployment.Wider reach

1 highlight

Implications of deployment.Cost

1 highlight

Overcoming barriers.Increase reach

1 highlight

Overcoming barriers.Financing

1 highlight

Overcoming barriers.Workforce

2 highlights

Overcoming barriers.Others

5 highlights

Overcoming barriers.Mobility

1 highlight

Role of UPDX CAD in Nigeria.Case detection

2 highlights

Role of UPDX CAD in Nigeria.Others

3 highlights

Role of UPDX CAD in Nigeria.Increases access

4 highlights

Role of UPDX CAD in Nigeria.Workforce

1 highlight
