# Supplementary material for: Operational feasibility of the ultra-portable digital X-rays with Computer-Aided Detection (CAD) for community active case finding for TB in Nigeria: Health care workers and client’s perspectives
Source: PLOS Glob Public Health. 2025 Oct 22;5(10):e0005234. doi: 10.1371/journal.pgph.0005234 (PMC12543118; doi:10.1371/journal.pgph.0005234)
Supplement: Attachment — Submitted filename: Response to Editorial Queries.docx [file pgph.0005234.s011.docx]

**RESPONSE TO QUERIES-PGPH-D-24-03076R1**

Operational feasibility of the Ultra-Portable digital X-rays with Computer-Aided Detection (CAD) for community active case finding for TB in Nigeria: Health care workers and client’s perspectives

Professor ATANA UKET EWA

Dear Jay-ar Formentera Medes,

1.    Figure 2: please (a) provide a direct link to the base layer of the map (i.e., the country or region border shape) and ensure this is also included in the figure legend; and (b) provide a link to the terms of use / license information for the base layer image or shapefile. We cannot publish proprietary or copyrighted maps (e.g. Google Maps, Mapquest) and the terms of use for your map base layer must be compatible with our CC-BY 4.0 license.

Note: if you created the map in a software program like R or ArcGIS, please locate and indicate the source of the basemap shapefile onto which data has been plotted. If your map was obtained from a copyrighted source please amend the figure so that the base map used is from an openly available source. Alternatively, please provide explicit written permission from the copyright holder granting you the right to publish the material under our CC-BY 4.0 license.

Please note that the following CC BY licenses are compatible with PLOS license: CC BY 4.0, CC BY 2.0 and CC BY 3.0, meanwhile such licenses as CC BY-ND 3.0 and others are not compatible due to additional restrictions. If you are unsure whether you can use a map or not, please do reach out and we will be able to help you. The following websites are good examples of where you can source open access or public domain maps:

* U.S. Geological Survey (USGS) - All maps are in the public domain. (http://www.usgs.gov)

* PlaniGlobe - All maps are published under a Creative Commons license so please cite “PlaniGlobe, http://www.planiglobe.com, CC BY 2.0” in the image credit after the caption. (http://www.planiglobe.com/?lang=enl)

* Natural Earth - All maps are public domain. (http://www.naturalearthdata.com/about/terms-of-use/)

Response: The map was created using a GIS software from GADM Maps and Data and edited using word paint. The link and source of the basemap shapefile is <https://gadm.org/maps/NGA.html>. The license information which allows for free use for academic publication is available on the website:

"GADM license

**The data are freely available for academic use and other non-commercial use. Redistribution or commercial use is not allowed without prior permission.**
Using the data to create maps for publishing of academic research articles is allowed. Thus you can use the maps you made with GADM data for figures in articles published by PLoS, Springer Nature, Elsevier, MDPI, etc. You are allowed (but not required) to publish these articles (and the maps they contain) under an open license such as CC-BY as is the case with PLoS journals and may be the case with other open access articles."

2.    Please upload all main figures as separate Figure files in .tif or .eps format only (individually). For more information about how to convert and format your figure files please see our guidelines:

<https://journals.plos.org/globalpublichealth/s/figures>

Response: Each figure file has been uploaded individually in .tif format. Thank you

3.    In the online submission form, you indicated that “Data availability statement: The raw data supporting the conclusions of this article is made available by the authors, without undue reservation”

All PLOS journals now require all data underlying the findings described in their manuscript to be freely available to other researchers, either

1. In a public repository,

2. Within the manuscript itself, or

3. Uploaded as supplementary information.

This policy applies to all data except where public deposition would breach compliance with the protocol approved by your research ethics board. If your data cannot be made publicly available for ethical or legal reasons (e.g., public availability would compromise patient privacy), please explain your reasons by return email and your exemption request will be escalated to the editor for approval. Your exemption request will be handled independently and will not hold up the peer review process, but will need to be resolved should your manuscript be accepted for publication. One of the Editorial team will then be in touch if there are any issues.

Your manuscript has been returned to your account. Please log on to PLOS Editorial Manager at <https://www.editorialmanager.com/pgph/> to access your manuscript.

Your manuscript can be found in the "Revisions Sent Back to the Author" link under the New Submissions menu. After you have made the changes requested above, please be sure to view and approve the revised PDF after rebuilding the PDF to complete the resubmission process.

Please note that these changes have been requested to comply with journal guidelines and your revised manuscript will *not* be sent to review until you have fully adhered to our requests. Once your paper has been seen by an Editor we may return it to you for further information or amendments.

Please resolve these issues and resubmit your manuscript within 30 days. If you need more time, please email the journal office at [globalpubhealth@plos.org.](mailto:globalpubhealth@plos.org.) We are happy to grant extensions of up to one month past this due date. If we do not hear from you within 30 days, we will withdraw your manuscript on your behalf.

Response: I had already uploaded all the raw data as supplementary files during the first submission and they are all found in the submission system. However, I will still re-submit the raw data files.

The terminology "Without undue reservation" actually means to do something completely and without any hesitation or doubt. That means I had submitted the raw data fully and unconditionally to Plos Global Public health, with no hidden doubts or conditions held back.

Thank you and best regards,

Professor Atana Uket Ewa

Corresponding author
